# The new holism: P4 systems medicine and the medicalization of health and life itself

**DOI:** 10.1007/s11019-016-9683-8

**Published:** 2016-01-28

**Authors:** Henrik Vogt, Bjørn Hofmann, Linn Getz

**Affiliations:** General Practice Research Unit, Department of Public Health and General Practice, Norwegian University of Science and Technology, Trondheim, Norway; Section for Health, Technology, and Society, Norwegian University of Science end Technology, Gjøvik, Norway; Centre for Medical Ethics, University of Oslo, Oslo, Norway

**Keywords:** Biomedicalization, Holism, Medicalization, P4 medicine, Personalized medicine, Precision medicine, Primary care, Quaternary prevention, Systems biology, Systems medicine

## Abstract

The emerging concept of systems medicine (or ‘P4 medicine’—predictive, preventive, personalized and participatory) is at the vanguard of the post-genomic movement towards ‘precision medicine’. It is the medical application of systems biology, the biological study of *wholes*. Of particular interest, P4 systems medicine is currently promised as a revolutionary new biomedical approach that is *holistic* rather than reductionist. This article analyzes its concept of *holism*, both with regard to methods and conceptualization of health and disease. Rather than representing a medical holism associated with basic humanistic ideas, we find a *technoscientific holism* resulting from altered technological and theoretical circumstances in biology. We argue that this holism, which is aimed at disease prevention and health optimization, points towards an expanded form of medicalization, which we call ‘*holistic medicalization*’: Each person’s whole life process is defined in biomedical, technoscientific terms as quantifiable and controllable and underlain a regime of medical control that is holistic in that it is *all*-*encompassing*. It is directed at all levels of functioning, from the molecular to the social, continual throughout life and aimed at managing the whole continuum from cure of disease to optimization of health. We argue that this medicalization is a very concrete materialization of a broader trend in medicine and society, which we call ‘*the medicalization of health and life itself*’. We explicate this holistic medicalization, discuss potential harms and conclude by calling for preventive measures aimed at avoiding eventual harmful effects of overmedicalization in systems medicine (quaternary prevention).

It is possible to get the life-phenomenon under our control … such a control and nothing else is the aim of biology—Biologist Jacques Loeb (1859–1924) (cited in Pauly 1987, p. 174).

At the risk of sounding academic, you are a system. A system made up of systems, to be exact. … Our integrative—or systems—approach holistically gathers, connects, and analyzes your data to create a complete picture of you, all 360 degrees of you ... At Arivale, we don’t guess. We base our recommendations—your roadmap—on your personal data story, that 360-degree view of you consisting of millions of data points.—The website of the P4 systems medicine company Arivale (2015).

## Introduction

This paper is motivated by authoritative claims that medicine, and especially primary care, will soon undergo a technologically driven system change associated with buzzwords such as ‘genomics’, ‘big data’, ‘digital health’ and ‘personalized’ or ‘precision medicine’ (ESF 2012; Topol 2012; Obama 2015). As a nexus where these developments come together we find the emerging field of *systems medicine* (see Table 1). Systems medicine is the medical application of *systems biology*, a 15-year old merger of molecular biology, mathematical modelling and systems theory (i.e. principles describing organized *wholes*) (O’Malley and Dupré 2005; De Backer et al. 2010; Bousquet et al. 2011; Green 2014). Systems medicine is often promoted as ‘*P4 medicine*’ (*predictive, preventive, personalized* and *participatory*). We will call it ‘*P4 systems medicine*’ (‘***P4SM***’).

**Table 1:** P4 systems medicine (predictive, preventive, personalized, participatory)

As shown by a PubMed search using the phrase ‘*systems medicine*’, this emerging biomedical concept is in rapid growth. For the year 2008 there were seven hits. By November 2015 there were approximately 400 hits for that year
*Research projects*: The Institute for Systems biology (ISB) recently launched the first phase (‘*The Hundred Person Wellness Project*’) of its ‘*100* *K Wellness Project*’. This is the first ‘real life’ clinical trial using P4SM principles. It is planned to involve quantification of a large number of parameters in 100,000 well people (Hood and Price 2014). In Europe the ‘Virtual Physiological Human’ and ‘Digital Patient’ are central research projects (Diaz et al. 2013; Hunter et al. 2013)
*Clinical reality*: P4SM is associated with concrete changes to clinical reality as highlighted in *the quantified self*-movement. Here individuals employ new technologies, for example genome sequencing and so-called ‘eHealth’, ‘mHealth’ or lifelogging tools (notably smart-phones) to continuously track their bodily functioning (Wolf 2009; Lupton 2014; Smarr 2012)
*Institutions and companies*: P4SM is associated with a range of research institutions in the USA, Europe and Asia. Systems medicine has gained support from the EU and European Commission, which have funded the ‘Coordinating Action Systems Medicine’ (CASyM) initiative to promote the implementation of systems medicine in Europe, as well as the Virtual Physiological Human and Digital Patient projects (Kirschner et al. 2013; Diaz et al. 2013; Hunter et al. 2013). In the USA, P4SM is strongly linked to the Institute for Systems Biology (ISB), the P4 medicine Institute (P4MI), the associated novel company Arivale. The latter has started to deliver actual health services. Another central US institution is the Harvard Medical School in Boston. In Europe, examples of central institutions are the European Institute for Systems Biology and Medicine (EISBM) in France, the Luxembourg Centre for Systems Biomedicine (LCSB), The University of Rostock, Germany and University College Dublin, Ireland. In Asia, there are institutions in India, Singapore and China, notably the Center for Systems Biomedicine in Shanghai

Crucially for this contribution, P4SM is associated with promises of a ‘*paradigm change*’ explained by the four Ps in ‘P4 medicine’ (Hood et al. 2012; Kirschner et al. 2013). Firstly, it promises a shift from a population-based ‘one-size-fits-all’ medicine to a ‘personalized (or ‘precision’) medicine’, which can account for the factors that define each particular individual (Duffy 2015). As shown in a recent textbook of personalized medicine, systems medicine overlaps with, and is having an increasing impact on, this wider concept and translational biomedical research in general (Jain 2015). Secondly, as a form of personalized medicine, it places particularly strong emphasis on shifting medicine from a focus on established disease to a prospective and proactive practice which focuses on *predictive* assessments of future health in order to facilitate disease *prevention* and *optimization* of health or wellness (‘health’ and ‘wellness’ are used synonymously here) (Diaz et al. 2013; Flores et al. 2013; Kirschner et al. 2013). Thirdly, according to its *participatory* aspect, it is promised to enable patients to shift to the role of agents driving the revolution.

Most importantly for our argument, P4SM is promised to achieve all this through a fourth change: A shift in biomedicine from a ‘*reductionist*’ towards a ‘*holistic*’ (or ‘*integrative*’^1^) approach, most vividly described by Vandamme et al. (2013):

In the medical practice, especially in that of the general practitioner, a more holistic, systems approach has always been used. The practitioner is confronted with the patient as a whole, and focuses on their individual needs and concerns. Every physician knows that each patient is different, that there is a need for a personalization of the medical treatment that they provide. He or she constantly has to try to integrate data on the emotional state of the patient, different comorbidities, environmental factors, family history, etc. In other words, physicians deal with a lot of non-linear, multidimensional information, while the medical science they need to use to make decisions provides them with tools to make linear, reductionist decisions. There is an overall theme of ‘one disease, one risk factor, one target’ with a lack of dynamic information. In the coming decade, systems medicine aims to provide the tools to take into account the complexity of the human body and disease in the everyday medical practice.

Such promises of holism may seem liberating to the humanistically minded medical generalist focusing on the health of the patient as a whole. However, at the same time, P4SM is evidently based on a *technoscientific* perspective^2^ that has often been at odds with such holism. As Galas and Hood (2009) state: ‘*Technology and new scientific strategies have always been the drivers of revolutions and this is certainly the case for P4 medicine*’.

### Our perspective

The authors of this paper write from the perspective of academic general practice, specifically a Nordic welfare state where primary health care is the organizational foundation. As the point of entry to medical care, it is specifically concerned with sustainable and responsible management of the health of whole persons over time (Getz 2006). The reason why we undertake an analysis of P4SM is that it explicitly aims to revolutionize the way primary care is provided, promising to tackle a range of its challenges, including waste and iatrogenic harm. And while some of its proponents have stated that academic medicine should ‘*lead, follow or get out of the way*’ (Snyderman and Yoediono 2008) as P4SM advances, we take a critical perspective.

### Aim and material

In light of the above, we aim to analyze the concept of *holism* in P4 systems medicine, both with regard to its methods and conceptualization of health and disease. We do not pretend to assess whether this is a ‘true’ holism or not, but describe its contents as presented in our material and some of its implications.

As our material we have selected a set of 40 publications comprehensively outlining P4SM as it stands today. Our scope is specific for P4SM, but we do see our analysis as relevant for understanding key developments in personalized medicine and international healthcare. For more on our material and scope, see endnotes.^3^^,^^4^

### Outline of the argument

Our main argument will be that P4SM aspires to make medicine holistic, yet does not entail holism as understood in what has been called *humanistic medicine*, a stream of medical thought and practice which focuses on the functioning, subjective experience and values of patients as whole persons, and which is frequently associated with an anti-medicalization stance (Engel 1977; Marcum 2008; McWhinney and Freeman 2009; Cassell 2013; Miles 2013). Contrary to this position, we observe how P4SM represents a *technoscientific holism* resulting from an altered, more all-encompassing technological gaze on human life and related changes in biomedicine’s methods and philosophy. We then argue that this form of holism points towards an expanded form of medicalization, which we call *holistic medicalization*: Each person’s whole dynamic life process is defined in biomedical, technoscientific terms as controllable and underlain a regime of control in terms of monitoring, quantification, prediction, risk profiling, early diagnosis, therapy, prevention and optimization that is *all*-*encompassing*. By ‘all-encompassing’ (which here corresponds to the term ‘holistic’) we mean *multi*-*dimensional, continual throughout life* as well as *directed at controlling all types of functioning, primarily healthy life*.

We do not by this pretend to discover an entirely new development. Rather, we argue that this expanded medicalization can be seen as the hitherto most concrete and comprehensive materialization of a broader trend, which has previously been described by several theorists and concepts. We will especially rely on three of these: *biohealth, biomedicalization and biopolitics* (Rose 2007; Clarke et al. 2010; Downing 2011). From our generalist point of view we see these concepts as closely related and refer to the historical development that they together describe as ‘*the medicalization of health and life itself*’. We here define *medicalization* very generally as *the process by which aspects of human life come to be defined in medical terms and underlain medical control* (Conrad 2007). We do not see this process as driven solely by medicine, but by many agents.

In the following, we will detail the above exposition in three parts. (1) In the first part, consisting of the sections ‘A technoscientific holism’ and ‘Health in technoscientific holism’, we show how the holism of P4SM arises from an interaction between theoretical and technological circumstances and specify how it defines health, disease and ‘life itself’. With regard to our argument, the main picture that emerges is that—however complex—these phenomena are rendered potentially *knowable* and *controllable* by biomedicine. (2) In the second part, consisting of the sections ‘Holistic medicalization’, ‘Holistic medicalization in practice’ and ‘Participatory medicalization’ we then spell out how this technoscientific holism points towards a *holistic medicalization*. (3) We then discuss implicated ‘Potential waste and harm’.

## A technoscientific holism

### A ‘holistic’ solution for biocomplexity

Aiming to analyze the meaning of ‘*holism*’ in P4 systems medicine, we will first explicate the historical context in which systems biologists use this term. In large part this is to contrast their approach to a personalized medicine based on the methods of molecular biology (Calvert and Fujimura 2009).

During the twentieth century there was always a stream of thought in biology stressing that living organisms are *more than the sum of their parts* and should be studied as integrated systems or *wholes* (Gilbert and Sarkar 2000). However, as tools for the scientific, empirical study of such wholes were largely unavailable, this *holism* was sidelined by the ‘*divide and conquer*’ strategy of molecular biology. Molecular biology can be said to have been ‘*reductionist*’ in that it was limited to focusing on one or a few, isolated bodily parts and relatively simple or *linear* causal relationships between parts (especially DNA) and the whole (health and disease). However, some 15 years after the sequencing of the human genome—molecular biology’s flagship project, culminating around the year 2000—this view is in crisis. Increasing empirical evidence has underscored that this genotype–phenotype relationship is more *complex* or *non*-*linear* than assumed (Woese 2004; Keller 2005). Systems medicine, which partly springs out of the human genome project and functional genomics, reflects this realization: In order to understand, predict—and thus *control*—the complexity of health and disease, one must study these phenomena in terms of integrated, dynamic, *complex systems* (Thomas 2007; Auffray et al. 2009; Antony et al. 2012; Wang et al. 2015). Systems medicine thus offers a solution to the challenge of biocomplexity that its proponents describe as ‘*holistic*’. Quote systems biologist Leroy Hood (2008):

The dominant challenge for all the scientific and engineering disciplines in the twenty-first century will be complexity, and biology is now in a unique position to solve the deep problems arising from its complexity and to begin to apply this knowledge to the most challenging issues of humankind. Biology will use systems approaches (holistic, as opposed to atomistic) and powerful new measurement and visualization technologies, as well as the new computational and mathematical tools that are emerging in the aftermath of the human genome project and the emergence of systems biology.

As indicated by this statement, a crucial enabling factor behind the holism of P4SM is new technology. These tools constrain the questions it may ask empirically. Crucially for our argument they also enable continued hope that, however complex, human wholes may yet be defined and controlled by science.

### A holistic method

Hood and Flores (2012) summarize the method of P4SM as follows:

Ironically many people use the term ‘genomic medicine’ to denote the medicine of the future—yet in principle genomic medicine is one-dimensional in nature—only encompassing nucleic acid information. Systems medicine, by contrast, is holistic and utilizes all types of biological information—DNA, RNA, protein, metabolites, small molecules, interactions, cells, organs, individuals, social networks and external environmental signals—integrating them so as to lead to predictive and actionable models for health and disease.

As exemplified by the above quotation, proponents of P4SM use the term ‘holistic’ in two related ways with regard to their methods and tools:

1. ‘*Holistic measurements*’: Firstly, the word ‘*holistic*’ comes to mean the use of new technologies to gather *big data* about each particular person that are as *all*-*encompassing* or ‘*global*’ as possible (De Backer et al. 2010; Diaz et al. 2013; Flores et al. 2013). These measurements in turn have two aspects that will reappear in what we call *holistic medicalization*: (a) Spatially, the measurements are *multi*-*dimensional* in that they pertain to all levels of biological organization. (b) Temporally, the technologies enable *repeated* or *continual* measurements through time that represent the dynamism of health and disease in a way that is new to biomedicine (Emmert-Streib and Dehmer 2013). The envisioned end result is a dynamic data cloud that reflects *the whole life process* in all four dimensions, consisting of ‘*billions of data points*’ (Bousquet et al. 2011).
  All conceivable technologies could potentially contribute to these measurements. However, the core data are molecular and enabled by new, high-throughput ‘*omics*’ technologies that generate whole ‘*parts lists*’ of molecules (e.g. genomics, proteomics, transcriptomics, metabolomics, epigenomics) (Ayers and Day 2015; Wang et al. 2015). Additionally, a massive phenotyping (’phenomics’) and mapping of environmental exposures is undertaken using for example microbiomics of bacterial flora, imaging, electronic health records, home telemonitoring, social media and various sensor technologies (implanted or external) coupled to smart-phones to monitor a range of bodily functions (Diaz et al. 2013).
2. ‘*Holistic (integrative) models*’: Secondly, the ‘*holistic*’ method involves using novel computer technologies to interpret the initially fragmented ‘*holistic data*’. One set of methods in this sense-making process comes from bioinformatics, but the key objective of systems medicine is to use mathematical modelling to *integrate* the data in what is called ‘*holistic multi*-*scale models*’ (Duffy 2015, see also Clermont et al. 2009; Wolkenhauer et al. 2014). According to Flores et al. (2013), ‘*These models decipher biological complexity by showing how all elements in biological systems interact with each other to produce health and disease states*’. A main goal is to study the way bodily systems transition between health and disease and thus generate the mechanistic explanations and predictive power needed to establish control of complex wholes (Hood and Price 2014). Crucially, the technologies of P4SM now also allow monitoring of the phases of life in which people are healthy, enabling nothing less than an attempt to *quantify health* (Hood 2013). This may be seen as an aspect of systems biology’s wider aim of *calculating life*, as expressed by Boogerd et al. (2007, chapter 14): ‘*With systems biology, life, first at the simplest level (…) and perhaps ultimately at the level of intelligent human beings will become calculable*’. Perhaps the ultimate expression of the goal of quantifying the whole life process is the European *Digital Patient* project, which aims to create a ‘*medical avatar*’ of each citizen to be compared to a generic ‘*virtual physiological human*’ (Hunter et al. 2013). According to its roadmap, ‘*Avatar literally means embodiment or manifestation and is a 4D personalised representation of individual patients*’ that can ‘*provide individualised (person*-*specific) future projections, systemic predictions based on mechanistic understanding of the disease process in an integrative and holistic view*’ (Diaz et al. 2013, p. 60 and p. 13).

### Holistic theory

On the theoretical level, the holism of P4SM corresponds to the idea of life as a complex system, which by definition refers to some kind of integrated whole. However, how a ‘*system*’ (and the *emergent properties* that arise from its dynamics) are understood may in turn vary, making different ‘holisms’ possible (O’Malley and Dupré 2005). Systems theory could potentially be used to argue that human health is so complex that it is hard to predict and control^5^ (Bishop 2011). However, in practice, the models that define P4SM theory seem mostly to be chosen with the aim of controlling the workings of wholes. As Tian et al. (2012) clearly state: ‘*Models may be descriptive, graphical or mathematical as dictated by the amount of available data, but they must be predictive. For medical use, predictions made must be actionable and useful for treating patients*’.

For this purpose models are mainly adopted from mathematics, physics, computer science and engineering (Antony et al. 2012; Hunter et al. 2013; Wolkenhauer et al. 2013; Wang et al. 2015). Moreno et al. (2011) describe this systems theory or ‘*network theory*’, as…

…the set of mathematical and computer simulation models and tools that have been developed to study network architectures and dynamics. Although there is no unified branch or corpus of mathematics that constitutes network theory, there exists however an increasingly indispensable ‘tool-kit’ of methods and disciplines that merge into what we might call network theory: this ranges from dynamical systems theory to network topology, from random boolean network models to coupled oscillators. The study of networks with strongly and recurrently interacting components allowed scientists to deal with holistic systems, showing that, despite their variety, they share certain generic properties.

In sum, we see a radically expanded approach to studying human beings.

## Health in technoscientific holism

This *technoscientific holism* alters biomedicine’s conception of health and disease (‘*the biomedical model*’) in several ways:

### Health as multi-level

**Fig. 1:**
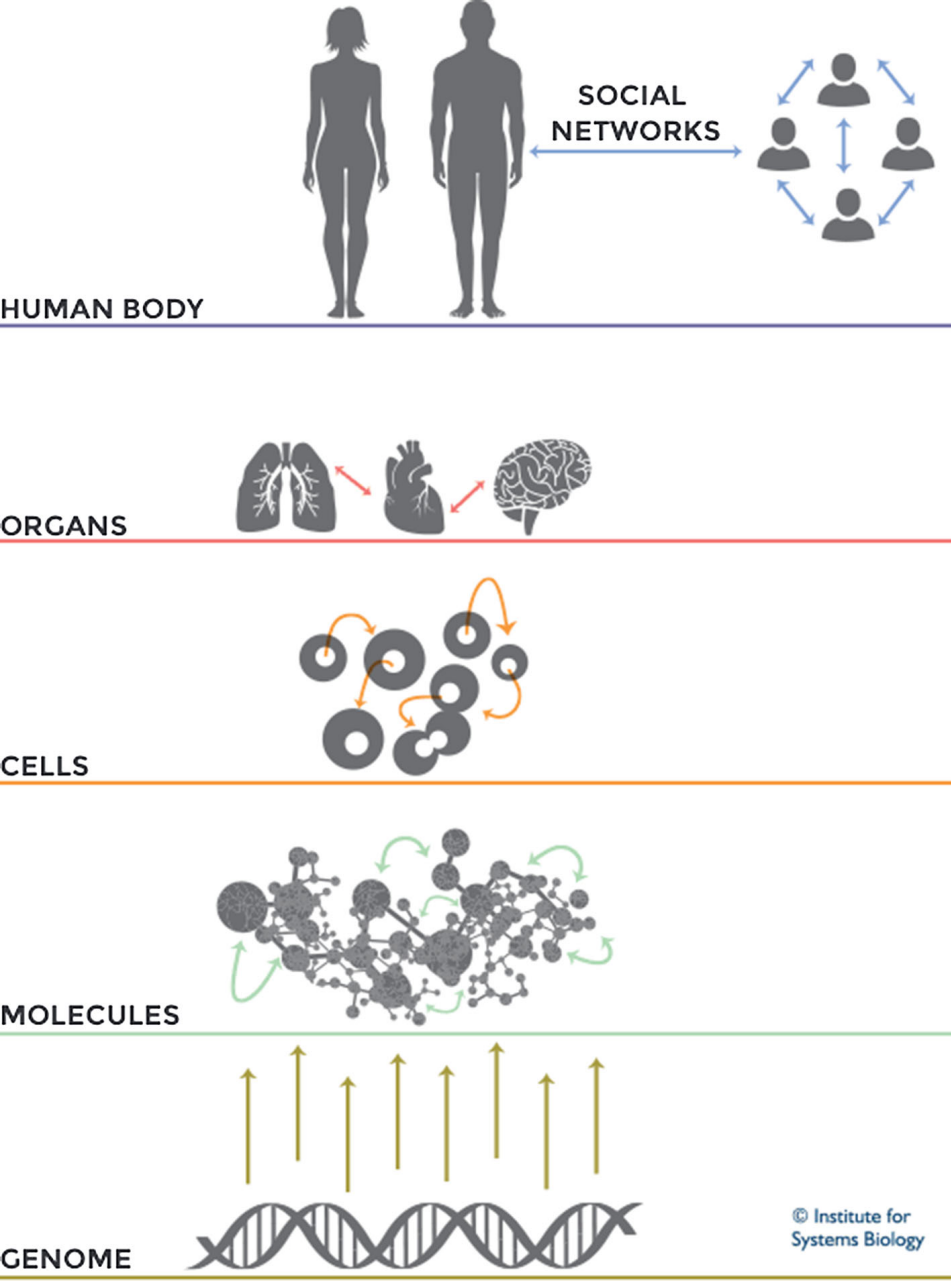
The human being as a dynamic network of networks. In systems medicine the human organism is envisioned as a system of systems or *network of networks*. At every scale of biological organization (molecular, cellular, organ, individual and social/environmental) systems are portrayed as giving rise to and embedding each other. At all levels the network of networks is seen as a dynamic or four-dimensional process (as opposed to a static thing) (Copyright: The Institute for Systems Biology, used with permission)

**Fig. 2:**
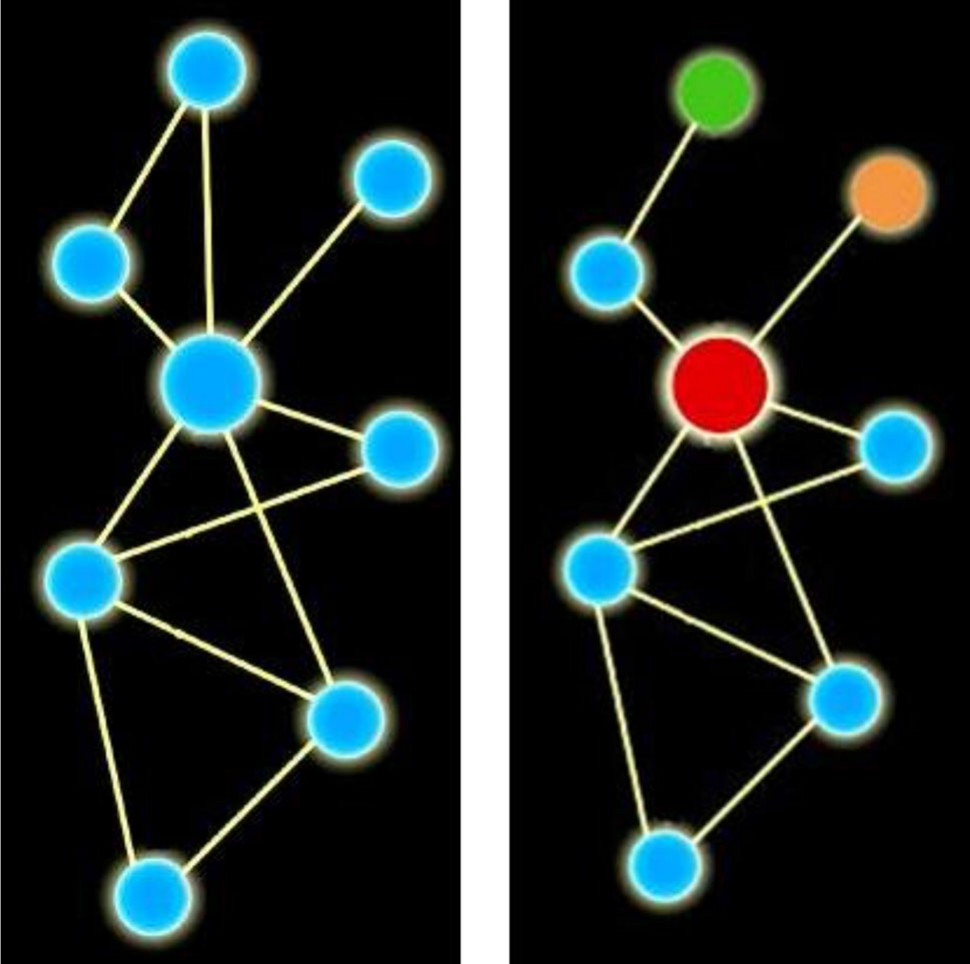
Disease and health as network states. One common way to represent systems or networks in PMSM is as *graphs*, where interacting units (e.g. molecules) are *nodes* and their interactions *edges*. According to one publication, this figure presents ‘A schematic view of a normal (*left*) and a disease-perturbed network (*right*). Both node points (*colored balls*) and edges (*lines attaching the balls*) change in disease as indicated by changing colors indicative of changing levels and the disappearance of an edge. The nodes and edges change dynamically with disease progression’ (Hood 2013).  (Copyright: The Institute for Systems Biology, used with permission). (Color figure online)

In P4SM technology and theory now allow health and disease to be characterized as multi-level phenomena. The whole human organism is portrayed as a highly non-linear system, often as a *network of networks* (Hood et al. 2012; Vandamme et al. 2013) (see Figs. 1, 2). P4SM thus takes biomedicine from conceptualizing health and disease as resulting from *linear relationships* between parts and wholes (a ‘*gene*-*centric*’ view) to a multi-causal, non-linear ‘*network*-*centric*’ view (Younesi and Hofmann-Apitius 2013).

### Health as process

Relatedly, P4SM’s longitudinal monitoring and theories of system dynamics promote a *process view* of health, taking biomedicine along an epistemological ‘*epigenetic turn*’ (Nicolosi and Ruivenkamp 2012). The development of each individual is conceptualized as the process of *gene*–*environment interaction (GxE),* where the ‘*environment*’ includes ‘psychosocial’ factors. Crucially, these interactions may be seen as genetic and environmental *information* that is integrated and encoded in the dynamic networks. To some systems biologists, biomedicine then becomes the informational science that deciphers this information (Bousquet et al. 2011).

### Health and disease as system states

This leads us to the P4SM definition of health and disease. Both phenomena are conceptualized as dynamic, functional *states* of the system that emerge through the GxE process (del Sol et al. 2010; Bousquet et al. 2011). These states may be seen as *emergent properties* of the whole, and health may also be conceptualized as *robustness* (the ability to maintain system integrity despite perturbations) (Federoff and Gostin 2009; Antony et al. 2012). In this picture, health and disease may also be seen as different aspects of a single *continuum* of potential network states in space and time (Hood and Flores 2012). Diseases may be defined in terms of *abnormal* and health in minimal terms as *normal network states* (del Sol et al. 2010) (see Fig. 2). The totality of possible states a system can be in is defined as a ‘*state space*’, and health and disease as different trajectories of states in this space (Antony et al. 2012).

### Health as individual specific

As the interactions between environmental and genetic factors are quite unique in each particular case, health and disease are underscored as individual-specific phenomena in P4SM. Technologies describing each individual in detail support this view. According to Bousquet et al. (2011), non-communicable diseases ‘*should be considered as the expression of a continuum or common group of diseases with intertwined gene*–*environment, socio*-*economic interactions and co*-*morbidities that lead to complex phenotypes specific for each individual.*’

### A mechanistic and predictable health

So far, we have described the factors that make the P4SM conception of health ‘*holistic*’. What qualifies this holism as ‘*technoscientific*’?

Systems biology is the site of deep epistemological and ontological discussions, notably about causation and predictability in living organisms (Wolkenhauer et al. 2013; Wolkenhauer and Green 2013; Boissel et al. 2015). However, mainstream P4SM seems to adhere to the machine metaphor of life. Mechanistic explanation and predictive power are main goals. Its mathematical models are often mechanistic and deterministic, and health and disease are widely defined as *mechanistically explainable.*^6^ Most significantly for our argument, the whole and its health are defined as potentially quantifiable (Antony et al. 2012; Diaz et al. 2013; Flores et al. 2013; Kirschner et al. 2013; Cesario et al. 2014b; Wang et al. 2015). In P4SM—as in systems biology—the concept of *holism* seems often to go hand in hand with the assumption that the emergent properties of the whole (i.e. health or illness) can be mechanistically explained and scientifically predicted (Boogerd et al. 2007). P4SM attempts to describe the unfolding process in mechanistic detail as something *concrete* (Boenink 2009). Consider Fig. 2: Represented in a technologically generated virtual reality, it is as if health and disease are objective and *there*. And while the metaphor of the genome as the ‘*book of life*’ may be obsolete, and the road towards unravelling the actual mechanistic workings of the body long, the whole life story may still be portrayed as *information* that biomedicine can decipher (Bousquet et al. 2011).

The most striking example of the idea of life as calculable is a programmatic proposition by leading figures to ‘*generate a multiparameter metric for wellness*—*by employing data from individuals exhibiting wellness over an extended period of time. It will reflect both the psychological and physiological aspects of wellness*’ (Hood et al. 2015a). According to Cesario et al. (2014b), ‘*Wellness as a status to be achieved and maintained in our lives, getting longer and hopefully healtier, is the new and comprehensive declination of* “*health*” *itself, leading the shaping of research and research policy in the health domain worldwide.*’ Flores et al. (2013) even state that P4SM models will be ‘*increasingly powerful predictors of each individual*’*s personal experience of health and disease. These models not only demystify disease, they also quantify what it means to be healthy*’.

All in all, the picture that emerges in P4SM is an effort to make medicine a *harder science* (Calvert and Fujimura 2009). It is a holism that entails a strong form of *naturalism*, which, in a medical context, is the view that health and disease are knowable through the methods of natural science (Marcum 2008).

### A controllable health

This naturalism is philosophically attractive: It appears to render not only disease but also states of wellness potentially *controllable.* Quote Hood and Flores (2012): ‘*Systems (P4) medicine is now pioneering something that never existed before*—*actionable understandings of disease and wellness as a continuum of network states, unique in time and space to each individual human being*’.

On the theoretical level, this is the essence of technoscientific holism: Although the whole life process of each individual is defined as complex, this whole—and the whole continuum of health and disease states it may be in—is defined as potentially quantifiable, predictable and *actionable*. ‘Actionable’ here means *controllable*. And defining an aspect of life in medical terms as controllable is a key step in the process of *medicalization*, leading us to the second main part of our argument (Conrad 2007).

## Holistic medicalization

We will now spell out how we see *technoscientific holism* as pointing towards a *holistic medicalization*. We begin by outlining the wider trend in which we place this development: ‘*the medicalization of health and life itself*’.

### The medicalization of health and life itself

Somewhat simplified, we can say that biomedicine, with its technoscientific approach, has traditionally been limited to a reactive and reductionist focus on parts-associated disease. As a corollary, biomedically rooted medicalization has mostly consisted in labelling aspects of life *asdiseases*. Healthy life has largely been dismissed as *the absence of disease* (Hofmann 2002; Marcum 2008). In other words, our path towards health itself has not been medicalized by technoscientific biomedicine like our approach to suffering.

The biomedical focus on disease has left an open space for attention to wholeness, health and human life-stories. Alternative medicine aside, this space has most often been filled by the holism of what has been called *humanistic medicine* (Marcum 2008). Such holism has been most strongly associated with medical generalism and concepts such as biopsychosocial, patient-centered, person-centered and narrative medicine (Engel 1977; McWhinney and Freeman 2009; Cassell 2013; Miles 2013). Traditionally, it is also this stream of thought that has advocated a shift in focus from disease towards the concept of health or well-being (Cassell 2013). Such holism has then typically linked health to capacities of *the whole person* as a conscious agent and tended to define these capacities as beyond exact scientific description. Instead, health has been tied to the most complex aspects of human life and thought to be a subjective and culturally value-laden (normative) phenomenon (Boenink 2009; Sturmberg 2013). Health has thereby been defined in positive terms as something *more and otherthan the absence of disease* that may even be *compatiblewith disease.* Critically, this form of holism has been associated with a certain tolerance towards disease and death and skepticism towards medicalization. Health has simply not been considered technoscientifically actionable like disease (Gadamer 1996; Hofmann 2002).

This traditional state of affairs is undergoing deep change through ‘the *medicalization of health and life itself*’ as described by three concepts: *biohealth*, *biomedicalization* and *biopolitics* (Rose 2007; Clarke et al. 2010; Downing 2011). With P4SM the undercurrents that these authors have pointed at seem to surface as a comprehensive framework.

### Biohealth, biomedicalization, biopolitics

Primary care physician and philosopher Raymond Downing describes his concept of *biohealth* as follows:

We are now in a phase beyond medicalization when even health—the “opposite” of medicine’s focus, disease—has become medicalized. Biomedicine, assuming it knows what health is, imposes that understanding on everyone. Medicine used to claim authority over the cracks and interruptions in life; now it claims authority over all of life (Downing 2011, p. 2).

According to Downing systems thinking inherently leads to an expansion of medicalization as it induces us to capture *all aspects* of a phenomenon (e.g. a person’s life). And: ‘*In describing or designing a system, we not only want to include every part, we also want to make each part captive, to control it*’ (Downing 2011, p. 70). Medicalization may in fact be seen as an inbuilt potential of all holism as holism is, by definition, *all*-*encompassing.*^7^

Similarly to *biohealth*, the concept of ‘*biomedicalization*’, developed by sociologist Adele Clarke and coworkers, describes an expansion of medicalization: A broad, multi-faceted trend towards not only defining and controlling evermore aspects of life *as disease*, but increasingly also towards *health optimization* and ‘*The extension of medical jurisdiction over health itself (in addition to illness, disease and injury*)’ (Clarke et al. 2010, p. 48).

*Biopolitics* (the term ‘politics’ connoting ‘power’) is a perspective developed by sociologist Nikolas Rose, building on philosopher Michel Foucault’s concept of *biopower*. It refers to *a management of life*, which…

…is neither delimited by the poles of illness and health, nor focused on eliminating pathology to protect the destiny of the nation. Rather, it is concerned with our growing capacities to control, manage, engineer, reshape, and modulate the very vital capacities of human beings as living creatures. It is, I suggest a politics of “life itself” (Rose 2007, p. 3).

### Holistic medicalization in theory

How does medicalization become ‘*holistic*’ on a theoretical level in P4SM; how does it *define aspects of life in medical terms*? Although this is not the place to enter the vast debates on health and holism, we first want to note that the concepts of *holism*, *wholes, wholeness* and *health* are etymologically and philosophically related. The very foundation of these ideas is now changing. ‘*Biohealth*’, Downing remarks is ‘*the sort of health and wholeness that results from applying the biological sciences*’ (Downing 2011, p. 6).

We will argue that the ‘*holistic medicalization*’ of P4SM is envisioned as the most advanced and systematic example to date of what Downing is pointing at. As we hope to have shown, the very idea of holism is redefined in P4SM and given a technoscientific meaning. In other words, the aspect of life that is defined in medical terms is *wholenessitself*. When the life process is understood in terms of dynamic wholes, but these wholes are defined as quantifiable and controllable through technoscientific means, biomedicalization becomes holistic on a theoretical level. *Holistic medicalization* does not primarily entail that aspects of life are defined *as new diseases*. Rather, it puts wholeness and health itself under medical jurisdiction, pointing toward a situation in which ‘life itself’ is controlled.

This is highlighted by the idea that one can provide *a multi*-*level metric for wellness*, with not only ‘*physiological*’, but also ‘*psychological*’ parameters by which to orient one’s way of life. According to this view, health is ‘*a concept that to date has been defined in vague and ambiguous terms*’ (Hood et al. 2015a). Through *technoscientific holism* this ‘*vagueness*’ is now to come to an end. As scientists at the Institute for Systems Biology state, ‘*wellness*—*and how to enhance it and extend it*—*has not been studied very thoroughly by scientists. ISB proposes to change this by taking a systems*-*approach to understanding wellness*—*and thereby make it scientific*’ (Hood et al. 2015b). This idea of a ‘*scientific wellness*’ becomes even more radical as the disease-health continuum should—at least according to Hood and coauthors—not be envisioned as stopping at *normality*, but as additionally involving a positive ‘*wellness space*’ of network states that is *more and other* than absence of disease (Flores et al. 2013). However, in sharp contrast to humanistic medical holism, see e.g. Cassell (2013), this positively defined health is portrayed as something that can be defined through technoscientific means.

### From molecularization towards synthesis

An important claim in the concepts of *biomedicalization* and *biopolitics* is that health and ‘life itself’ have increasingly become *molecularized* (Rose 2007; Clarke et al. 2010). Rose remarks that ‘*It is now at the molecular level that human life is understood, at the molecular level that its processes can be anatomized, and at the molecular level that life can now be engineered*’ (Rose 2007, p. 4). Does the holism of P4SM also involve such *molecularization*? The answer is ‘*yes, but…*’.

Systems biology is technologically strongly focused on molecular parts (De Backer et al. 2010). As a corollary the wholes that P4SM models are also mostly *molecular wholes* (Mardinoglu and Nielsen 2012; Emmert-Streib and Dehmer 2013; Ayers and Day 2015; Wang et al. 2015). As illustrated by talk of ‘*molecular level (…) processes that define and drive physiology*’ and provide ‘*deep understanding of causality*’ (Flores et al. 2013), the molecular is central to its philosophy of causation and epistemology. The overall aim is to connect the whole to its parts, and the whole seems mostly to be defined *in molecular terms*. Such a *molecular holism* is a contradiction in terms and better understood as a form of *reductionism* (De Backer et al. 2010).

However, at the same time, P4SM involves profound discussions about the organizing principles by which wholes govern parts (Antony et al. 2012; Wolkenhauer et al. 2013; Wolkenhauer and Green 2013). Its deepest theoretical contribution may be that it slowly moves biomedicine from a one-dimensional molecular focus towards a view where no level is causally or epistemologically privileged (Noble 2007; O’Malley and Dupré 2005). Quote Boissel et al. (2015): ‘*The solution must include multilevel interactions in an integrative approach. Thus, systems medicine should go beyond the realm of the intracellular layer to integrate upper physiological layers, including all time and complexity level components*’.

This seems partly at odds with Rose’s view of molecularization as an epistemological change away from the nineteenth century view of the body as a ‘*system of systems*’ (Rose 2007, p. 43). P4SM represents precisely a move towards such a view. Our key point in this article is that this also changes medicalization. As new tools now seem to allow synthesis, the process of medicalization also moves beyond molecularization to a *synthetic phase*. The endgame of medicalization will result from trying to piece all bodily pieces together to define and control life *in toto*.

### Defining the limits of medicalization

In one publication, Juengst et al. (2012) voiced concerns over potential problems with personalized medicine—for example pursuit of human enhancement. P4SM proponents responded by pointing precisely to their *holism*:

For the most part, these concerns are alleviated by eliminating the undue focus on genetics (…), the scientific and technological foundation of P4 healthcare rests on systems approaches to big data on many different dimensions of health, not just genomic factors. Systems approaches are powerful precisely because they integrate all of these data to delineate how environmental and genetic factors interact to shape individual experiences of health and disease (Flores et al. 2013).

However, to the extent that medicalization poses problems, this argument offers cold comfort. For the more *holistic* P4SM will become, while at the same time defining ‘*the whole*’ as technoscientifically actionable, the more medicalizing it may get. The perfect endgame, a technologically based mirror image—or ‘*avatar*’—of each individual that enables the prediction and control of all health aspects—will logically also involve *total medicalization*. We conjecture that systems medicine will define the future limits of medicalization. As the major rate-limiting hindrance to medicalization is biocomplexity, and P4SM models will likely define biomedicine’s uttermost efforts to overcome this complexity, it will also delineate the degree to which life can be technoscientifically controlled.

## Holistic medicalization in practice

What would holistic medicalization of P4SM look like in practice, if realized? A vision presented in the European ‘*Digital Patient*’ roadmap is illuminating:

The vision of a “digital me” that contains all my health-care information, (…) communicated with all my wearable and implanted technology to constantly monitor my health status and informing me, my family and friends, or my healthcare providers of alarming events, supporting the collaboration of various specialists around my complex systemic diseases, and used with all my data to predict the future development of my health in order to facilitate disease prevention and a fully self-aware lifestyle (Diaz et al. 2013, p. 57).

Firstly, we see that P4SM would still be directed at managing disease, especially chronic disease that requires long-term management of life (Cesario et al. 2014a). However, while disease would still be one focus, healthcare would shift in scope so as to favour the management of the health and lives of healthy or asymptomatic people—that is *all people.* This management would be both multi-level, continuous throughout the lifecourse and directed at all types of network states all along the continuum from overt pathology, via more or less well-discerned risk profiles, ‘normality’ and into the positive ‘*wellness space*’ (Diaz et al. 2013; Flores et al. 2013).

The main thrust in P4SM would be *prevention* involving risk profiling and early diagnosis in healthy or asymptomatic people (Bousquet et al. 2011). The definition of health as potentially predictable and controllable may be key to this ambition:

In the future, we will be able to design drugs to prevent networks from becoming disease perturbed. For example, if there is an 80 % change of prostate cancer at age 50, taking these preventive drugs beginning at age 35 may reduce disease probability to 2 % (Hood 2008).

In addition to disease management and prevention, P4SM practice would—at least according to some leading proponents—not only be directed at disease as something negative, but *optimization* of *health* or *wellness* as something positive. Another ‘P’ could thereby be added to ‘P4’: Promotive medicine. According to Boissel et al. (2015), ‘*The optimization of wellness is a key to maximizing human potential for each individual*—*improving physiological as well as psychological performances.*’

With regard to such optimization, one key aim is to elucidate and manipulate the process of ageing, which is hard to delineate from the process of ‘life itself’ (Hood and Flores 2012; Bousquet et al. 2014). As an example, the *Digital Patient Roadmap* reduces aging to the dominant risk factor of disease: ‘*Ageing is a hurdle to overcome and its inclusion is personalised models for the Digital Patient is a challenge that multi*-*scale models will need to resolve*’ (Diaz et al. 2013). Even more profoundly, systems biology is also the basis of *synthetic biology*, which aims specifically to engineer new properties of life (Auffray et al. 2009).

Optimization or *enhancement* of human capacities is central to the concepts of *biohealth, biomedicalization* and *biopolitics* (i.e. the’ medicalization of health and life itself’). According to Rose, ‘*The old lines between treatment, correction, and enhancement can no longer be sustained*’ (Rose 2007, p. 6). When P4SM becomes sufficiently efficient in transforming the processes of life, these borders will blur. In the P4SM visions, prevention also seems based on optimization, and optimization always to imply that something is in some way *suboptimal*. It also seems clear that the proposed P4SM metrics of health will involve many of the same (predominantly molecular) parameters used to define disease (Wang et al. 2010). If so, the language of health will to a large extent be derived from the language of disease.

### Diagnostics and prognostics

As part of a preventive and health-optimization strategy, diagnostics and prognostics would as default involve a multi-level, continuous and individualized monitoring or *screeningprocess* (Bousquet et al. 2011). This amounts to an advanced form of what has been called *surveillance medicine* (Armstrong 1995). Although the diagnostic process would use information from all levels in a ‘holistic’ fashion, it would be biomarker-based (Mardinoglu and Nielsen 2012; Younesi and Hofmann-Apitius 2013). And while previous biomarkers have mostly been single-component, future P4SM biomarkers may be ‘*network biomarkers*’ (Wang et al. 2015). One aim is to make ‘*blood a window for assessing health and disease*’ (Hood and Flores 2012) by constructing diagnostic technologies that can regularly assess ‘*molecular fingerprints*’ reflecting specific network states (Wang et al. 2010).

Risk is a key concept in *the medicalization of health and* ‘*life itself*’ (Clarke et al. 2010; Downing 2011; Rose 2007). With regard to disease prevention, we conjecture that the very concepts of risk or susceptibility to disease will change in P4SM. As the idea of what holds our destiny changes from static DNA or a few riskfactors to the workings of the dynamic network, they will become more multi-factorial and dynamic concepts. The need to account for all these factors implies a increased focus on risk that is unprecedented in its all-encompassing scope.

If P4SM would venture into active health optimization, ‘a*ctionable possibilities*’ would additionally emerge in positive *wellness space*—pointing perhaps towards a radically new *diagnostics of health* (Hood and Price 2014). Preliminary results from the ISB ‘*Hundred Person Wellness Project*’ show how expansive P4SM may become in labelling well people: ‘*So far, after having analyzed just a few types of data, we*’*ve found that 100* *% of the 100 Pioneers have multiple actionable possibilities*’ (Hood 2014). Here, the concept of ‘an actionable’, frequently used by Hood and collegues, may both be understood as a relabelling of the traditional concept of risk of disease, but also as a piece of information that may be useful in enhancing one’s wellness or performance.

### Intervention

Like the diagnostic process, intervention would also turn into a life-long, dynamic project that would be directed at tackling all components of the complexity of health, including potentially the personal (or ‘psychological’) and social. A large group of P4SM advocates argues that management of non-communicable disease (NCD)…

…should move towards holistic multi-modal integrated care, and multi-scale, multi-level systems approaches. (…) Systems medicine aims to tackle all components of the complexity of NCDs so as to understand these various phenotypes and hence enable prevention (Bousquet et al. 2011).

Genes are no longer regarded as destiny. As a corollary, P4SM emphasises preventive lifestyle interventions (Bousquet et al. 2014; Hood 2014). In this regard, what is considered ‘medical treatment’ might potentially change and focus more on non-technological intervention. This may also seem *non*-*medicalizing*. However, biomedicine would still strengthen its grip on what it means to lead a healthy life, and even lifestyle, living itself, would be grounded in a continuous, technologically based monitoring of risk-factors.

In practice, operationalizing and modelling complex personal and social factors is harder and has a much lower priority in current P4SM research than the molecular level. Drug development is a main focus: ‘*By deciphering which biological networks are perturbed in diseases, systems medicine will provide a stream of new drug targets for the pharmaceutical industry*’ (Flores et al. 2013). In accordance with the principles of network theory, pharmaceutical intervention would also change, turning into a process of control engineering. As the view of what must be controlled changes from linear to non-linear, treatment is also envisioned as multicausal: ‘*A new “network*-*centric” rather than “gene*-*centric” approach to choosing drug targets will employ multiple drugs to “re*-*engineer” a disease*-*perturbed network to make it behave in a more normal manner*’ (Hood and Flores 2012). In other words, technoscientific holism leads to a complexification of pharmaceutical treatment and polypharmacy as default.

## Participatory medicalization

According to its *participatory* aspect, P4SM is envisioned as requiring an expansion of healthcare far beyond the clinic. Patients, families and communities working in networks are expected to drive its realization (Diaz et al. 2013; Hood and Auffray 2013; Kirschner et al. 2013). As this aspect defines P4SM, it will also define an aspect of *holistic medicalization*. We will call this *participatory medicalization*.

Again the novel technologies of P4SM are the enabling factor. Firstly, they are primarily directed at the individual body and its subsystems. Correspondingly, both personal *and* public health is tied to the individual person, who is expected to live life to the fullest in symbiosis with the biomedical tools that provide access to health. The longitudinal cloud of billions of data points gathered for each individual allows his/her life to be envisioned as a form of *N*-*of*-*1 study* in which each person is a vital participant in his or her own ‘holistic’ description (Kirschner et al. 2013; Hood and Price 2014). This is one hallmark of ‘the medicalization of health and life itself’: It is focused on the individual and health becomes a personal goal and responsibility (Rose 2007; Clarke et al. 2010; Downing 2011).

Secondly, the quest for *personalized* medicine (somewhat paradoxically) requires *everyone* to participate. To develop valid predictive power, P4SM needs data from a population that is as big and diverse as possible in order to mine the data for regularities, to demarcate health and to stratify the population:

In order to take into account the full range of biological complexity and define the range of healthy behavior, these data need to be obtained for as many people as possible in the population—ideally everyone—not just for small test samples (Flores et al. 2013).

This need also explains *imperatives* for people to share their data:

We stress that patients must understand that it is their societal responsibility to make their anonymized data available to appropriate scientists and physicians so that the latter can create the predictive medicine of the future that will transform the health of their children and grandchildren (Bousquet et al. 2011).

In other words: Participation is a requirement for the holism of P4SM and the holistic medicalization it implies. It involves what Rose calls *a mode ofsubjectification*…

…through which individuals are brought to work on themselves, under certain forms of authority, (…) by means of practices of the self, in the name of their own life or health, that of their family or some other collectivity, or indeed in the name of the life or health of the population as a whole (Rabinow and Rose 2006).

The participation of each patient consumer in novel social communities is a vivid example of what Rose’s collaborator, anthropologist Paul Rabinow, terms *biosociality* (Rabinow and Rose 2006).

As a pioneering example of the wellness-regime that P4SM hopes to establish, computer scientist Larry Smarr has published results from 10 years of self-monitoring (Smarr 2012; Hood and Price 2014). Smarr employed multiple tools to measure his genome, blood markers (>100 variables), stool markers, diet, exercise, sleep and stress, pointing also towards more ‘*wholesome*’ personal omics profiling in future *self*-*quantification*.

With reference to the *quantified self*-*movement* that Smarr pioneered (Table 1), we may predict that P4SM will contribute strongly to what Rose calls ‘*biological citizenship*’ or ‘*somatic individuality*’, the formation of a kind of personal identity in which ‘*we are increasingly coming to relate to ourselves as “somatic” individuals (…) who experience, articulate, judge, and act upon ourselves in part in the language of biomedicine*’ (Rose 2007, p. 26). As an enabling factor of this development, some technologies (e.g. tracking devices) or at least their results (e.g. genome information) are becoming cheaply available to citizens. Technology is *democratized.* As a consequence, P4SM is presented as *democratizing* and *empowering,* enabling people to *know themselves* and establish *self*-*control* (Hood and Price 2014; Duffy 2015). However, to the extent that people will gain—or lose—genuine control of their life, they will do so according to the metrics of P4SM. To be in a position to define a metric of health according to which people manage their lives is power, an example of what Rose calls ‘*somatic expertise*’ and *ethopolitics*, the latter meaning ‘*attempts to shape the conduct of human beings by acting upon their sentiments, beliefs, and values*—*in short by acting on their ethics*’ through ‘*self*-*techniques by which human beings should judge and act upon themselves to make themselves better than they are*’ (Rose 2007, p. 27).

We thus argue that what is most evidently ‘*democratized*’ in participatory medicine is the ability to *self*-*medicalize*, and in P4SM more ‘*holistically*’ than ever. Patients may become more active, but their goals are still defined by the agents behind P4SM. It should be noted that the leaders of the ‘*Hundred Person Wellness Project*’ project (see Table 1) recently stated that most of its participants ‘*established a new and very personalized baseline for their own health*’ through the research (Hood et al. 2015b). However, it is unclear at this point how each individual’s personal baseline of health is thought established (e.g. is it defined using measures of subjective well-being or molecular markers?). It is also unclear how population-based metrics of health and each person’s baseline are to relate (e.g. which one of them will actually define what health means in the individual case?).

## Potential waste and harm

### Control as value and goal

The extent to which health can in practice be given a meaningful scientific definition and controlled is an open question. However, one does not have to actually *succeed* in controlling human wholes for *holistic medicalization* to be realized. One only needs to *believe* it possible and make the attempt. As evident in the *quantified self*-movement, the values and goals of P4SM are likely to become defining to identities and actions in healthcare and beyond even before the framework is supported by empirical evidence (Lupton 2014; Wolf 2009; Smarr 2012). What are the values and goals of P4SM? Manifold, but we will state just one: *Control itself*. Physiologist Claude Bernard (1813–1878), who foresaw the application of mathematics to biology and has been called ‘*the first systems biologist*’, can also serve as an historical reference for this ideal (Noble 2007). While Bernard had sophisticated ideas of living wholes, he also stated that ‘*When an experimenter succeeds in learning the necessary conditions’ of the phenomena he is studying*, “*he is, in some sense, its master; he can predict its course and appearance, he can promote or prevent it at will*” (cited in Comfort 2012, p. 46). Consider also our opening quote by Loeb, who pioneered the biological engineering ideal so prevalent in P4SM. We see P4SM as biomedicine’s latest and most all-encompassing step in pursuing this goal.

### Beneficent and harmful medicalization

That said we want to stress that we see nothing inherently wrong with control or medicalization. Beneficent control is a key aim of medicine. However, as reflected in the ancient medical proverb ‘*first do no harm*’, all medicalization also comes with caveats (i.e. ‘*overmedicalization*’ or ‘*futile medicalization*’). A full discussion of all caveats implicated by the holistic medicalization of P4SM is beyond our scope, but we will point out what we find most fundamental.

### False positives, overdiagnosis, opportunity cost

Firstly, the number of measurements and continuous (self)-management of well people will likely increase findings of uncertain significance, false positive tests, overdiagnosis and overtreatment (Diamandis 2015). Even if these problems were nullified, the sheer amount of work done by all involved agents would represent a significant *distraction* of attention and economic resources away from other problems and solutions that also matter in life (opportunity cost).

### Social and cultural iatrogenesis

The most insidious danger, however, may be what Ivan Illich termed *social* and *cultural iatrogenesis* (Illich 1975): It may lead to a damaging labelling of aspects of life *as medical* and displace other valid goals, values and ways of understanding and tackling life. Biomedicalization may distort our understanding of problems that should be understood on the personal, social or political levels by describing them in reductive biological terms. The holism of humanistic medicine has traditionally considered health a phenomenon that is hard to separate from ‘*the good life*’ itself (Hofmann 2002; Boenink 2009). When previous definitions of health have been deliberately ‘vague’, it is precisely because this phenomenon is exceedingly complex, an *enigma* defying any simple attempt at a definition (Gadamer 1996). In accounting for all its critical aspects, P4SM can never be truly *holistic* or *person*-*specific*. Like all science it must necessarily involve reduction and generalization (Vogt et al. 2014; Wolkenhauer 2014). If P4SM insists that it can eradicate the ‘*vagueness*’ of health, it also risks denigrating the ‘*the good life*’ by ignoring what its metrics cannot capture. As Downing states of ‘*biohealth*’ (Downing 2011, p. 6).

Health means wholeness; qualifying it by “bio-” narrows it down to a certain sort of wholeness, that which is brought about by the application of the biological sciences. Those applications may be very beneficial, but those benefits cannot be called health, because they are not whole.

Critically, while the holism of what we have called humanistic medicine has focused on what is *good enough* in life and exercised a certain tolerance towards disease, we cannot find the possibility of health being *compatible with disease* mentioned in our material. Even more profoundly, the fact that everyone eventually grows frail and dies—and how to handle this—is completely absent from P4SM as a proposed framework. As evident in one of its policy requirements, P4SM is instead associated with *perfectionism*: ‘*Set a benchmark for the U.S. to become the “healthiest nation”, like putting a man on the moon*’ (Hood and Galas 2008). Evidently, P4SM risks creating illness-generating and cost-increasing expectations of wellness (Callahan 1998).

### Narratives versus bio-narratives

Our species is biologically defined by an ability to generate meaningful stories that define our lives and sense of health (Cassell 2013). What P4SM promises to do through its continual monitoring and modelling is to redefine such life stories as what we may call technoscientfically constituted *bio*-*narratives*. In a very real sense, it amounts to a new ‘*bio*-*narrative medicine*’, promising literally ‘*to develop a series of stories about how actionable opportunities have changed the wellness of the participants*’ (Hood et al. 2015b). Such *bio*-*narratives* may potentially help document the importance of personal experience and agency, but they may also displace other narratives. Consider remarks made by researchers of the ‘*Hundred Person Wellness Project*’:

Almost all individuals came to the study with the view that they were (for the most part) well. However, the study exposed for all individuals multiple actionable possibilities that could be acted upon to improve their wellness. This illustrated that most of us have unrealized potential for optimizing our wellness (Hood et al. 2015b).

These people entered the clinic feeling healthy, but according to their *bio*-*narrative*—as interpreted—they ‘*in fact, have multiple abnormalities in biochemical markers reflecting organ and system dysfunction, nutritional status or other health risk*’ (Hood et al. 2015a). In this case, each participant’s experience of health seems trumped by P4SM metrics. There is no scientific reason, however, why the *bio*-*narrative* should be privileged in defining health. This is, at least in part, a conceptual question.

### The last well person

Hood and coworkers have argued that damaging effects from risk information and positive findings is ‘*a myth*’ (Hood et al. 2015a). Rather than going into an empirical discussion about this disputable conclusion, we will make a philosophical argument that *holistic medicalization* must by necessity have major disruptive effects on human life. As philosopher Hans-Georg Gadamer puts it, ‘*health itself*’ is the ability to ‘*forget that one is healthy*’ (Gadamer 1996, p vii). This would seem impossible in P4SM. What is at stake is no less than a person’s own ability to state ‘*I am well*’ without having to consult a computational mirror image. In a 1994 article in The New England Journal of Medicine, M.D. Clifton K. Meador satirically predicted the demise of what he called ‘*The Last Well Person*’, noting that ‘*Well people are disappearing.* (…) *I began to realize what was happening only a year ago, at a dinner party. Everyone there had something*’ (Meador 1994). If P4SM defines 100 % of the population with something ‘*actionable*’, it is risks fulfilling Meador’s prophecy.

## Conclusion

We have argued that what we have called the technoscientific holism of P4SM points towards a ‘*holistic medicalization*’, to date the most systemetic and comprehensive expression of a broader ‘*medicalization of health and life itself*’ that may also define the limits of medicalization in the future. With P4SM the ‘*divide and conquer*’ of previous reductionist biomedicine is replaced by ‘*synthesize and conquer*’. It moves from hoping to control disease by manipulating of a few factors to hoping to control it through management of the whole, dynamic life process. This is not a return to the holism of humanistic medicine, as in medicine that is focused on the defining capacities, subjective experience and values of whole persons. Rather, it is biopsychosocial, patient-centered and person-centered medicine—or the ‘*art*’ of medicine—being redrawn in technoscientific terms.

Despite launching an unprecedented expansion of medicalization, P4SM advocates have not yet engaged in judicious discussions of potential downsides. We therefore want to conclude by affirming that its *holism* calls for *quaternary prevention*. Quaternary prevention is a growing thrust in preventive medicine aiming to ‘*Reduce overmedicalization (overdiagnosis and overtreatment) and iatrogenic harm*’ through ‘*action taken to protect individuals (persons/patients) from medical interventions that are likely to cause more harm than good*’ (Brodersen et al. 2014). The words of biologist Carl Woese (2004) calling for *a new biology for a new century* also seem relevant for health care and preventive measures in the coming years: ‘*A society that permits biology to become an engineering discipline, that allows that science to slip into the role of changing the living world without trying to understand it, is a danger to itself*’.

## Footnotes

1 In systems biology the term ‘integrative’ may be used interchangeably with ‘holistic’. ‘Integrative biology’ is a synonym for ‘systems biology’ and the moniker ‘integrative healthcare’ may overlap with ‘systems medicine’ although it is also used in alternative medicine (Jain 2015).

2 In this article the term ‘*technoscientific*’ is used broadly to refer to any phenomenon that is at once scientific and strongly linked to technology.

3 This article is part of a larger project investigating P4SM as a proposed framework for primary care, and as such it is based on a thorough research into the subject matter and underlying philosophical issues involving many literature searches. To delineate a suitable and transparent material for this analysis, we performed the following PubMed search: ‘“systems medicine” OR “systems biomedicine” OR “P4 medicine” OR “4P medicine”’. Last performed 2015-11-23 this search yielded 1623 hits. We focused on identifying comprehensive, theoretical publications outlining P4SM that are broad in scope and especially relevant for understanding P4SM as a framework for primary care. We excluded shorter publications (e.g. editorials) and publications focusing on particular medical problems or specialities. We thereby selected 31 publications, the oldest from 2008. We see this material as reflecting the emerging field of P4SM well. As described by other authors, systems biology may be divided in different schools (O'Malley and Dupré 2005; De Backer et al. 2010; Thomas 2007). The dominant “pragmatic” or “molecular” school is rooted in functional genomics and has traditionally sought network-based explanations at the cellular level using high-throughput ‘omics’ data. There is also a school rooted in traditional physiology, which is more focused on models connecting all biological levels. A ‘systems-theoretical’ school describes researchers that focus strongly on systems theory principles. These schools are now merging and all are reflected in our material. However, it suitably mostly reflects the dominant school. The most influential research environment here is located at The Institute for systems biology (ISB) in Seattle, USA. Its leading figure, Leroy Hood, is represented in 10 of the 31 initial publications. Seeing its ‘*Hundred Person Wellness Project*’ (see Table 1) as especially relevant for our article, we searched the ISB’s list of publications and annual reports at www.systemsbiology.org and identified 5 additional publications. Based on our research (reading of reference lists etc.) we also identified and included an early online document, a European ‘Roadmap to Systems medicine’, a European ‘Digital Patient Roadmap’ as well as a strategy publication for the Virtual physiological human (Hood and Galas 2008; Kirschner et al. 2013; Hunter et al. 2013; Diaz et al. 2013). The latter two are representative of the ‘physiological’ school of systems medicine. In sum, we included 40 publications, all identified in our reference list.

4 P4SM cannot be seen as representative of biomedicine at large. However, it is relevant for understanding of broad developments in international healthcare. Recent remarks made by US president Barack Obama announcing the Precision Medicine Initiative may serve as an example (Obama 2015): “*The (…) human genome (…) today (…) costs less than $2000. Wearable electronics make it easier than ever to record vital signs (…) And more powerful computers help us analyze data faster than ever before. So if we combine all these emerging technologies, if we focus them and make sure that the connections are made (…) the possibilities are boundless*.’ Obama also emphasised participatory medicine (‘…*we want every American ultimately to be able to securely access and analyze their own health data, so that they can make the best decisions for themselves and for their families*’). As another example, British Secretary of Health, James Hunt, recently declared: ‘*If you talk to technology gurus in California (…) They say “You can get three hundred thousand biomarkers from a single drop of blood, so why would you depend on a human brain to calculate what that means when a computer can do it for you*? *(…) I think it’s really important that we’re ready in the NHS to harness the power of data to give us more accurate diagnoses (…) What this will mean, is we can identify problems before they’re symptomatic and therefore have a much better chance of tackling them*’ (Matthews-King 2015). These comments are related to the ideas of P4SM. Moreover, one may note that professor Eric Topol, author of the ‘*The Creative Destruction of medicine*’, is in the scientific advisory board at the P4SM company Arivale (see Table 1). P4SM also influences US academic medicine (Snyderman and Yoediono 2008).

5 Here it seems pertinent to note that, in the past, representatives of humanistic medicine have also employed systems theory in their holistic conceptions of health, but then mainly to bolster an emphasis on the social interactions and personal experience of human wholes (Engel 1977; McWhinney and Freeman 2009; Sturmberg 2013).

6 A radio analogy of life has been frequently used in systems biology. Leroy Hood brings it to systems medicine: ‘*A holistic, integrative or systems approach can be explained by a simple analogy. In order to understand how a radio converts electromagnetic waves into sound waves, the first step would be to compile a list of its components. Then the components would be studied individually to ascertain what each component does independently. After understanding the individual parts, the next step would be to assemble the parts into circuits and then understand individually and collectively how the circuits convert radio waves into sound waves*’ (Hood 2013).

7 Downing also directs the criticism that systems thinking leads to medicalization towards the use of systems theory in Engel’s biopsychosocial model and patient-centered medicine (Engel 1977; McWhinney and Freeman 2009). However, we see important differences between this and P4SM. The adoption of systems theory in what has been called *humanistic medicine* has been intended as a metaphorical tool when one competent clinician seeks to ‘model’ other persons in order to avoid reducing their problems to molecules. In P4SM, such ‘human modelling’ would be replaced mathematical modelling and the doctor-patient relationship with a computer interface and computational decision support.
